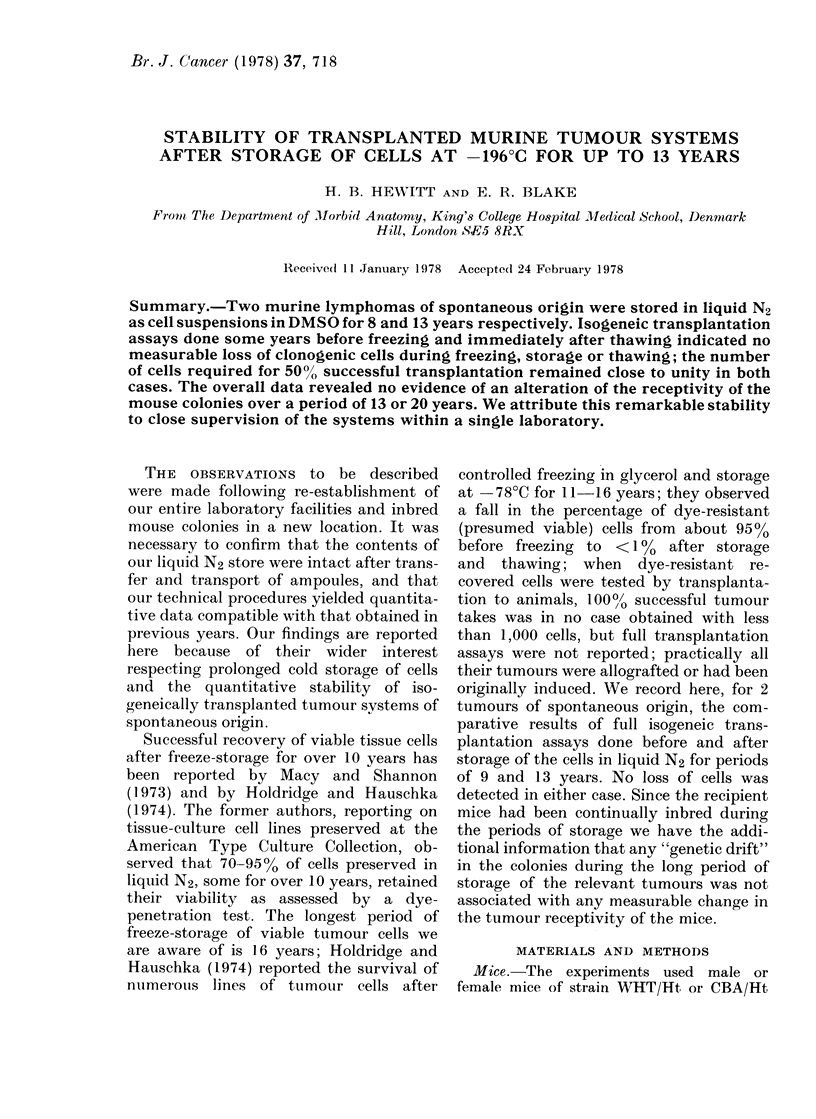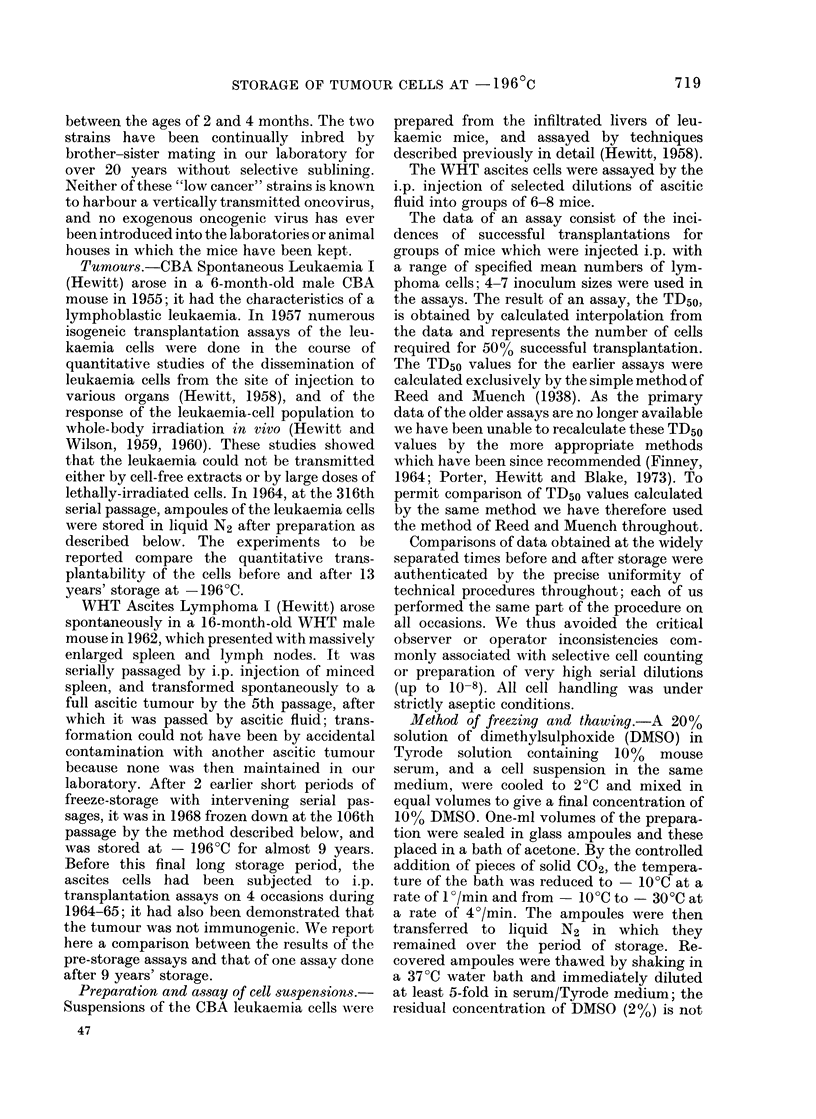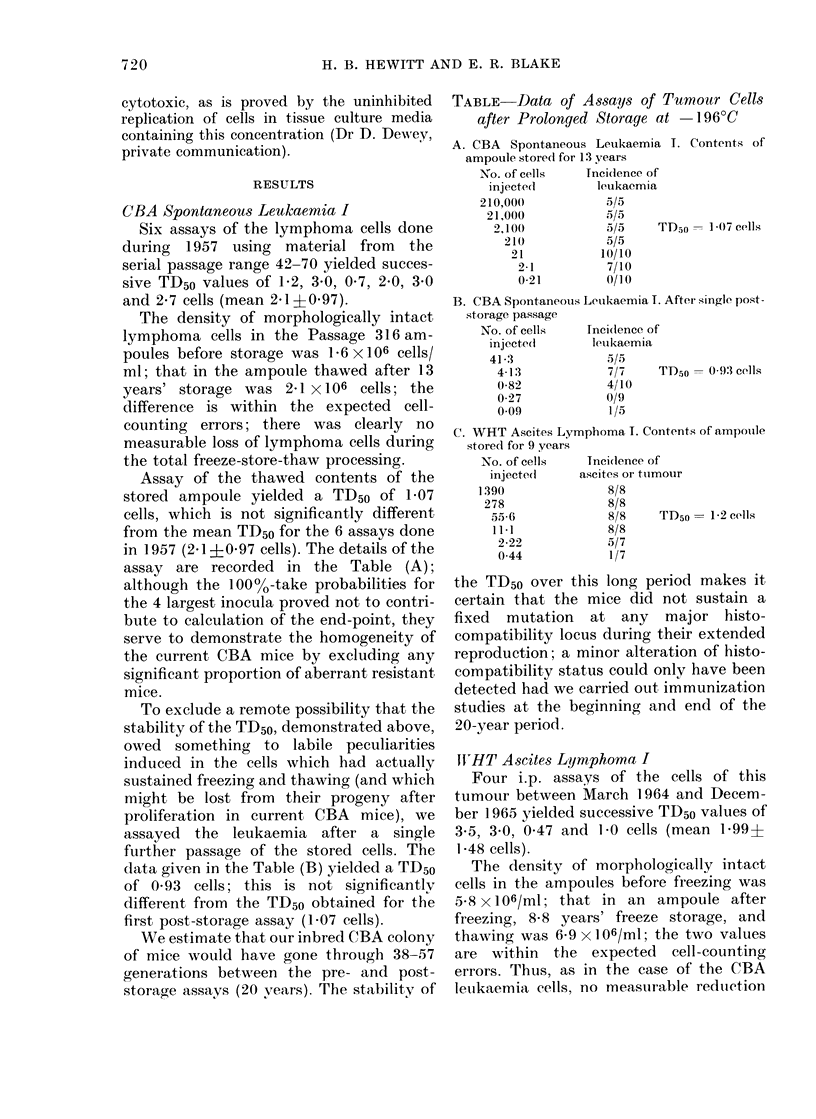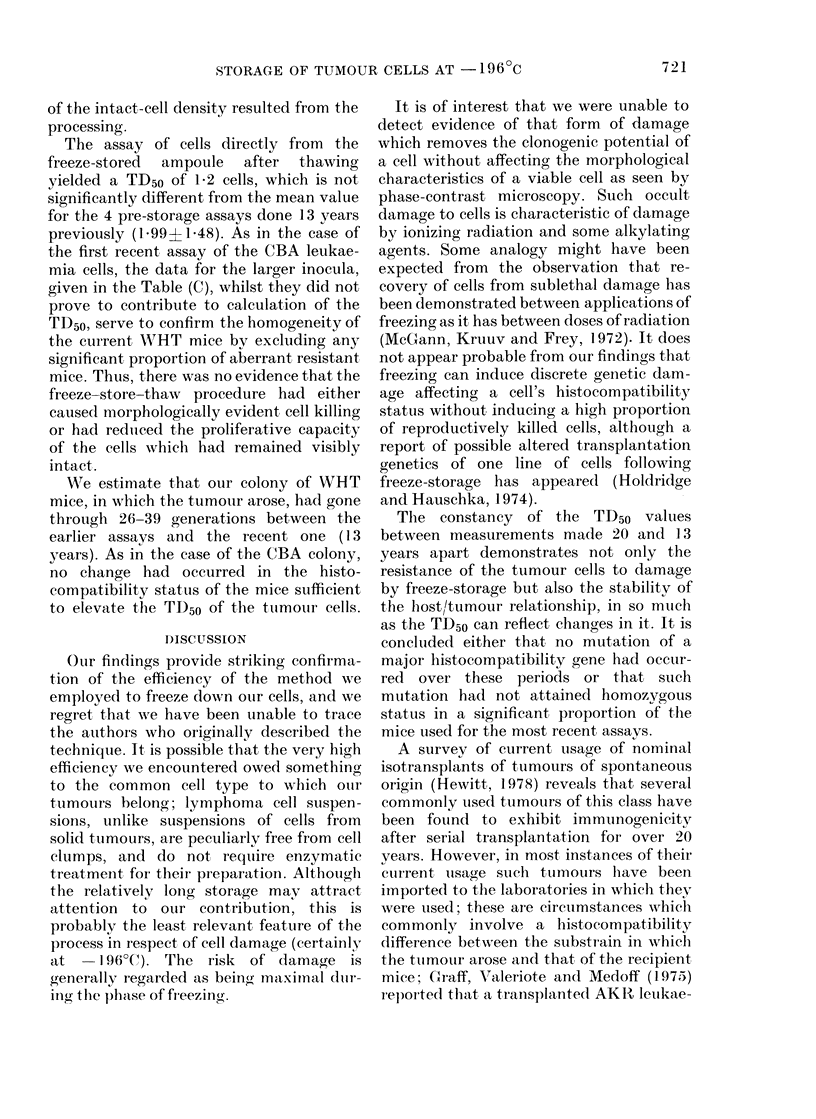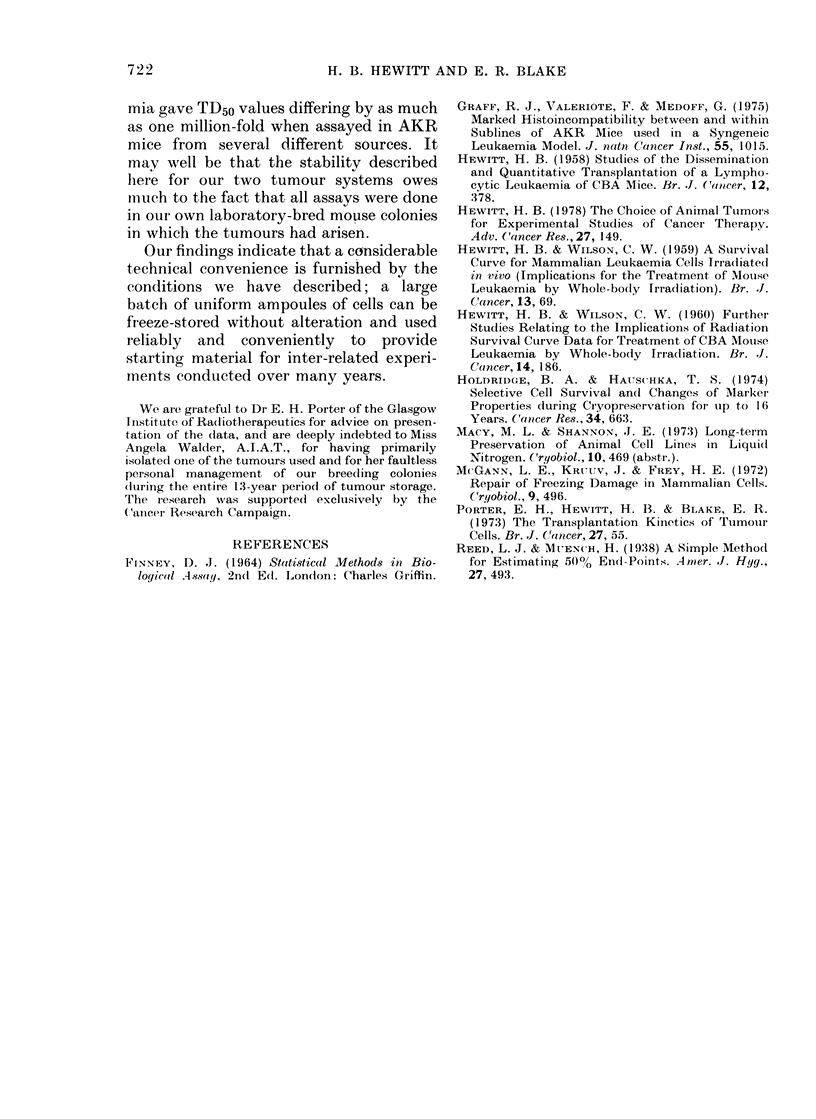# Stability of transplanted murine tumour systems after storage of cells at -196 degrees C for up to 13 years.

**DOI:** 10.1038/bjc.1978.109

**Published:** 1978-05

**Authors:** H. B. Hewitt, E. R. Blake

## Abstract

Two murine lymphomas of spontaneous origin were stored in liquid N2 as cell suspensions in DMSO for 8 and 13 years respectively. Isogenic transplantation assays done some years before freezing and immediately after thawing indicated no measurable loss of clonogenic cells during freezing, storage or thawing; the number of cells required for 50% successful transplantation remained close to unity in both cases. The overall data revealed no evidence of an alteration of the receptivity of the mouse colonies over a period of 13 or 20 years. We attribute this remarkable stability to close supervision of the systems within a single laboratory.


*Br. J. Cancer* (1978) **37**, 718

### STABILITY OF TRANSPLANTED MURINE TUMOUR SYSTEMS AFTER STORAGE OF CELLS AT −196°C FOR UP TO 13 YEARS

H. B. HEWITT AND E. R. BLAKE

*From The Department of Morbid Anatomy, King's College Hospital Medical School, Denmark Hill, London SE5 8RX*

Received 11 January 1978 Accepted 24 February 1978

**Summary.—Two murine lymphomas of spontaneous origin were stored in liquid $N_2$ as cell suspensions in DMSO for 8 and 13 years respectively. Isogeneic transplantation assays done some years before freezing and immediately after thawing indicated no measurable loss of clonogenic cells during freezing, storage or thawing; the number of cells required for 50% successful transplantation remained close to unity in both cases. The overall data revealed no evidence of an alteration of the receptivity of the mouse colonies over a period of 13 or 20 years. We attribute this remarkable stability to close supervision of the systems within a single laboratory.**

THE OBSERVATIONS to be described were made following re-establishment of our entire laboratory facilities and inbred mouse colonies in a new location. It was necessary to confirm that the contents of our liquid $N_2$ store were intact after transfer and transport of ampoules, and that our technical procedures yielded quantitative data compatible with that obtained in previous years. Our findings are reported here because of their wider interest respecting prolonged cold storage of cells and the quantitative stability of isogeneically transplanted tumour systems of spontaneous origin.

Successful recovery of viable tissue cells after freeze-storage for over 10 years has been reported by Macy and Shannon (1973) and by Holdridge and Hauschka (1974). The former authors, reporting on tissue-culture cell lines preserved at the American Type Culture Collection, observed that 70–95% of cells preserved in liquid $N_2$, some for over 10 years, retained their viability as assessed by a dye-penetration test. The longest period of freeze-storage of viable tumour cells we are aware of is 16 years; Holdridge and Hauschka (1974) reported the survival of numerous lines of tumour cells after controlled freezing in glycerol and storage at −78°C for 11—16 years; they observed a fall in the percentage of dye-resistant (presumed viable) cells from about 95% before freezing to <1% after storage and thawing; when dye-resistant recovered cells were tested by transplantation to animals, 100% successful tumour takes was in no case obtained with less than 1,000 cells, but full transplantation assays were not reported; practically all their tumours were allografted or had been originally induced. We record here, for 2 tumours of spontaneous origin, the comparative results of full isogeneic transplantation assays done before and after storage of the cells in liquid $N_2$ for periods of 9 and 13 years. No loss of cells was detected in either case. Since the recipient mice had been continually inbred during the periods of storage we have the additional information that any "genetic drift" in the colonies during the long period of storage of the relevant tumours was not associated with any measurable change in the tumour receptivity of the mice.

### MATERIALS AND METHODS

*Mice.*—The experiments used male or female mice of strain WHT/Ht or CBA/Ht between the ages of 2 and 4 months. The two strains have been continually inbred by brother–sister mating in our laboratory for over 20 years without selective sublining. Neither of these "low cancer" strains is known to harbour a vertically transmitted oncovirus, and no exogenous oncogenic virus has ever been introduced into the laboratories or animal houses in which the mice have been kept.

*Tumours.*—CBA Spontaneous Leukaemia I (Hewitt) arose in a 6-month-old male CBA mouse in 1955; it had the characteristics of a lymphoblastic leukaemia. In 1957 numerous isogeneic transplantation assays of the leukaemia cells were done in the course of quantitative studies of the dissemination of leukaemia cells from the site of injection to various organs (Hewitt, 1958), and of the response of the leukaemia-cell population to whole-body irradiation *in vivo* (Hewitt and Wilson, 1959, 1960). These studies showed that the leukaemia could not be transmitted either by cell-free extracts or by large doses of lethally-irradiated cells. In 1964, at the 316th serial passage, ampoules of the leukaemia cells were stored in liquid $N_2$ after preparation as described below. The experiments to be reported compare the quantitative transplantability of the cells before and after 13 years' storage at $-196^{\circ}C$.

WHT Ascites Lymphoma I (Hewitt) arose spontaneously in a 16-month-old WHT male mouse in 1962, which presented with massively enlarged spleen and lymph nodes. It was serially passaged by i.p. injection of minced spleen, and transformed spontaneously to a full ascitic tumour by the 5th passage, after which it was passed by ascitic fluid; transformation could not have been by accidental contamination with another ascitic tumour because none was then maintained in our laboratory. After 2 earlier short periods of freeze-storage with intervening serial passages, it was in 1968 frozen down at the 106th passage by the method described below, and was stored at $-196^{\circ}C$ for almost 9 years. Before this final long storage period, the ascites cells had been subjected to i.p. transplantation assays on 4 occasions during 1964–65; it had also been demonstrated that the tumour was not immunogenic. We report here a comparison between the results of the pre-storage assays and that of one assay done after 9 years' storage.

*Preparation and assay of cell suspensions.*—Suspensions of the CBA leukaemia cells were prepared from the infiltrated livers of leukaemic mice, and assayed by techniques described previously in detail (Hewitt, 1958).

The WHT ascites cells were assayed by the i.p. injection of selected dilutions of ascitic fluid into groups of 6–8 mice.

The data of an assay consist of the incidences of successful transplantations for groups of mice which were injected i.p. with a range of specified mean numbers of lymphoma cells; 4–7 inoculum sizes were used in the assays. The result of an assay, the $TD_{50}$, is obtained by calculated interpolation from the data and represents the number of cells required for 50% successful transplantation. The $TD_{50}$ values for the earlier assays were calculated exclusively by the simple method of Reed and Muench (1938). As the primary data of the older assays are no longer available we have been unable to recalculate these $TD_{50}$ values by the more appropriate methods which have been since recommended (Finney, 1964; Porter, Hewitt and Blake, 1973). To permit comparison of $TD_{50}$ values calculated by the same method we have therefore used the method of Reed and Muench throughout.

Comparisons of data obtained at the widely separated times before and after storage were authenticated by the precise uniformity of technical procedures throughout; each of us performed the same part of the procedure on all occasions. We thus avoided the critical observer or operator inconsistencies commonly associated with selective cell counting or preparation of very high serial dilutions (up to $10^{-8}$). All cell handling was under strictly aseptic conditions.

*Method of freezing and thawing.*—A 20% solution of dimethylsulphoxide (DMSO) in Tyrode solution containing 10% mouse serum, and a cell suspension in the same medium, were cooled to $2^{\circ}C$ and mixed in equal volumes to give a final concentration of 10% DMSO. One-ml volumes of the preparation were sealed in glass ampoules and these placed in a bath of acetone. By the controlled addition of pieces of solid $CO_2$, the temperature of the bath was reduced to $-10^{\circ}C$ at a rate of $1^{\circ}$/min and from $-10^{\circ}C$ to $-30^{\circ}C$ at a rate of $4^{\circ}$/min. The ampoules were then transferred to liquid $N_2$ in which they remained over the period of storage. Recovered ampoules were thawed by shaking in a $37^{\circ}C$ water bath and immediately diluted at least 5-fold in serum/Tyrode medium; the residual concentration of DMSO (2%) is not cytotoxic, as is proved by the uninhibited replication of cells in tissue culture media containing this concentration (Dr D. Dewey, private communication).

### RESULTS

### *CBA Spontaneous Leukaemia I*

Six assays of the lymphoma cells done during 1957 using material from the serial passage range 42–70 yielded successive $TD_{50}$ values of 1·2, 3·0, 0·7, 2·0, 3·0 and 2·7 cells (mean $2{\cdot}1 \pm 0{\cdot}97$).

The density of morphologically intact lymphoma cells in the Passage 316 ampoules before storage was $1{\cdot}6 \times 10^6$ cells/ml; that in the ampoule thawed after 13 years' storage was $2{\cdot}1 \times 10^6$ cells; the difference is within the expected cell-counting errors; there was clearly no measurable loss of lymphoma cells during the total freeze-store-thaw processing.

Assay of the thawed contents of the stored ampoule yielded a $TD_{50}$ of 1·07 cells, which is not significantly different from the mean $TD_{50}$ for the 6 assays done in 1957 ($2{\cdot}1 \pm 0{\cdot}97$ cells). The details of the assay are recorded in the Table (A); although the 100%-take probabilities for the 4 largest inocula proved not to contribute to calculation of the end-point, they serve to demonstrate the homogeneity of the current CBA mice by excluding any significant proportion of aberrant resistant mice.

To exclude a remote possibility that the stability of the $TD_{50}$, demonstrated above, owed something to labile peculiarities induced in the cells which had actually sustained freezing and thawing (and which might be lost from their progeny after proliferation in current CBA mice), we assayed the leukaemia after a single further passage of the stored cells. The data given in the Table (B) yielded a $TD_{50}$ of 0·93 cells; this is not significantly different from the $TD_{50}$ obtained for the first post-storage assay (1·07 cells).

We estimate that our inbred CBA colony of mice would have gone through 38–57 generations between the pre- and post-storage assays (20 years). The stability of the $TD_{50}$ over this long period makes it certain that the mice did not sustain a fixed mutation at any major histocompatibility locus during their extended reproduction; a minor alteration of histocompatibility status could only have been detected had we carried out immunization studies at the beginning and end of the 20-year period.

TABLE—*Data of Assays of Tumour Cells after Prolonged Storage at* $-196°C$

A. CBA Spontaneous Leukaemia I. Contents of ampoule stored for 13 years

| No. of cells injected | Incidence of leukaemia | |
|---|---|---|
| 210,000 | 5/5 | |
| 21,000 | 5/5 | |
| 2,100 | 5/5 | $TD_{50}$ = 1·07 cells |
| 210 | 5/5 | |
| 21 | 10/10 | |
| 2·1 | 7/10 | |
| 0·21 | 0/10 | |

B. CBA Spontaneous Leukaemia I. After single post-storage passage

| No. of cells injected | Incidence of leukaemia | |
|---|---|---|
| 41·3 | 5/5 | |
| 4·13 | 7/7 | $TD_{50}$ = 0·93 cells |
| 0·82 | 4/10 | |
| 0·27 | 0/9 | |
| 0·09 | 1/5 | |

C. WHT Ascites Lymphoma I. Contents of ampoule stored for 9 years

| No. of cells injected | Incidence of ascites or tumour | |
|---|---|---|
| 1390 | 8/8 | |
| 278 | 8/8 | |
| 55·6 | 8/8 | $TD_{50}$ = 1·2 cells |
| 11·1 | 8/8 | |
| 2·22 | 5/7 | |
| 0·44 | 1/7 | |

### *WHT Ascites Lymphoma I*

Four i.p. assays of the cells of this tumour between March 1964 and December 1965 yielded successive $TD_{50}$ values of 3·5, 3·0, 0·47 and 1·0 cells (mean $1{\cdot}99 \pm 1{\cdot}48$ cells).

The density of morphologically intact cells in the ampoules before freezing was $5{\cdot}8 \times 10^6$/ml; that in an ampoule after freezing, 8·8 years' freeze storage, and thawing was $6{\cdot}9 \times 10^6$/ml; the two values are within the expected cell-counting errors. Thus, as in the case of the CBA leukaemia cells, no measurable reduction of the intact-cell density resulted from the processing.

The assay of cells directly from the freeze-stored ampoule after thawing yielded a $TD_{50}$ of 1·2 cells, which is not significantly different from the mean value for the 4 pre-storage assays done 13 years previously (1·99±1·48). As in the case of the first recent assay of the CBA leukaemia cells, the data for the larger inocula, given in the Table (C), whilst they did not prove to contribute to calculation of the $TD_{50}$, serve to confirm the homogeneity of the current WHT mice by excluding any significant proportion of aberrant resistant mice. Thus, there was no evidence that the freeze–store–thaw procedure had either caused morphologically evident cell killing or had reduced the proliferative capacity of the cells which had remained visibly intact.

We estimate that our colony of WHT mice, in which the tumour arose, had gone through 26–39 generations between the earlier assays and the recent one (13 years). As in the case of the CBA colony, no change had occurred in the histocompatibility status of the mice sufficient to elevate the $TD_{50}$ of the tumour cells.

### DISCUSSION

Our findings provide striking confirmation of the efficiency of the method we employed to freeze down our cells, and we regret that we have been unable to trace the authors who originally described the technique. It is possible that the very high efficiency we encountered owed something to the common cell type to which our tumours belong; lymphoma cell suspensions, unlike suspensions of cells from solid tumours, are peculiarly free from cell clumps, and do not require enzymatic treatment for their preparation. Although the relatively long storage may attract attention to our contribution, this is probably the least relevant feature of the process in respect of cell damage (certainly at —196°C). The risk of damage is generally regarded as being maximal during the phase of freezing.

It is of interest that we were unable to detect evidence of that form of damage which removes the clonogenic potential of a cell without affecting the morphological characteristics of a viable cell as seen by phase-contrast microscopy. Such occult damage to cells is characteristic of damage by ionizing radiation and some alkylating agents. Some analogy might have been expected from the observation that recovery of cells from sublethal damage has been demonstrated between applications of freezing as it has between doses of radiation (McGann, Kruuv and Frey, 1972). It does not appear probable from our findings that freezing can induce discrete genetic damage affecting a cell's histocompatibility status without inducing a high proportion of reproductively killed cells, although a report of possible altered transplantation genetics of one line of cells following freeze-storage has appeared (Holdridge and Hauschka, 1974).

The constancy of the $TD_{50}$ values between measurements made 20 and 13 years apart demonstrates not only the resistance of the tumour cells to damage by freeze-storage but also the stability of the host/tumour relationship, in so much as the $TD_{50}$ can reflect changes in it. It is concluded either that no mutation of a major histocompatibility gene had occurred over these periods or that such mutation had not attained homozygous status in a significant proportion of the mice used for the most recent assays.

A survey of current usage of nominal isotransplants of tumours of spontaneous origin (Hewitt, 1978) reveals that several commonly used tumours of this class have been found to exhibit immunogenicity after serial transplantation for over 20 years. However, in most instances of their current usage such tumours have been imported to the laboratories in which they were used; these are circumstances which commonly involve a histocompatibility difference between the substrain in which the tumour arose and that of the recipient mice; Graff, Valeriote and Medoff (1975) reported that a transplanted AKR leukaemia gave $TD_{50}$ values differing by as much as one million-fold when assayed in AKR mice from several different sources. It may well be that the stability described here for our two tumour systems owes much to the fact that all assays were done in our own laboratory-bred mouse colonies in which the tumours had arisen.

Our findings indicate that a considerable technical convenience is furnished by the conditions we have described; a large batch of uniform ampoules of cells can be freeze-stored without alteration and used reliably and conveniently to provide starting material for inter-related experiments conducted over many years.

We are grateful to Dr E. H. Porter of the Glasgow Institute of Radiotherapeutics for advice on presentation of the data, and are deeply indebted to Miss Angela Walder, A.I.A.T., for having primarily isolated one of the tumours used and for her faultless personal management of our breeding colonies during the entire 13-year period of tumour storage. The research was supported exclusively by the Cancer Research Campaign.

### REFERENCES

FINNEY, D. J. (1964) *Statistical Methods in Biological Assay*, 2nd Ed. London: Charles Griffin.

GRAFF, R. J., VALERIOTE, F. & MEDOFF, G. (1975) Marked Histoincompatibility between and within Sublines of AKR Mice used in a Syngeneic Leukaemia Model. *J. natn Cancer Inst.*, **55**, 1015.

HEWITT, H. B. (1958) Studies of the Dissemination and Quantitative Transplantation of a Lymphocytic Leukaemia of CBA Mice. *Br. J. Cancer*, **12**, 378.

HEWITT, H. B. (1978) The Choice of Animal Tumors for Experimental Studies of Cancer Therapy. *Adv. Cancer Res.*, **27**, 149.

HEWITT, H. B. & WILSON, C. W. (1959) A Survival Curve for Mammalian Leukaemia Cells Irradiated *in vivo* (Implications for the Treatment of Mouse Leukaemia by Whole-body Irradiation). *Br. J. Cancer*, **13**, 69.

HEWITT, H. B. & WILSON, C. W. (1960) Further Studies Relating to the Implications of Radiation Survival Curve Data for Treatment of CBA Mouse Leukaemia by Whole-body Irradiation. *Br. J. Cancer*, **14**, 186.

HOLDRIDGE, B. A. & HAUSCHKA, T. S. (1974) Selective Cell Survival and Changes of Marker Properties during Cryopreservation for up to 16 Years. *Cancer Res.*, **34**, 663.

MACY, M. L. & SHANNON, J. E. (1973) Long-term Preservation of Animal Cell Lines in Liquid Nitrogen. *Cryobiol.*, **10**, 469 (abstr.).

McGANN, L. E., KRUUV, J. & FREY, H. E. (1972) Repair of Freezing Damage in Mammalian Cells. *Cryobiol.*, **9**, 496.

PORTER, E. H., HEWITT, H. B. & BLAKE, E. R. (1973) The Transplantation Kinetics of Tumour Cells. *Br. J. Cancer*, **27**, 55.

REED, L. J. & MUENCH, H. (1938) A Simple Method for Estimating 50% End-Points. *Amer. J. Hyg.*, **27**, 493.